# Trends, hotspots, and future directions of inflammation in age-related macular degeneration: A 20-year bibliometric study

**DOI:** 10.1097/MD.0000000000046598

**Published:** 2026-03-20

**Authors:** Fangfang Lu, Jia Zhang, Jing Lu, Weijie Jiang, Zhigang Fei, Qiguo Xiao, Zhi Wang

**Affiliations:** a Department of Ophthalmology, The Second Affiliated Hospital, Hengyang Medical School, University of South China, Hengyang, Hunan, China.

**Keywords:** age-related macular degeneration, bibliometric analysis, CiteSpace, inflammation, VOSviewer

## Abstract

**Background:**

Inflammation significantly impacts the pathogenesis of age-related macular degeneration (AMD), the primary cause of vision loss in the elderly. With the aging global population and increasing incidence of AMD, understanding the inflammation-related mechanisms and identifying potential therapeutic targets have become paramount. This study aims to offer a bibliometric analysis of global research contributions focused on inflammation in AMD, shedding light on research trends, collaborations, and future directions.

**Methods:**

We retrieved publications related to inflammation in AMD from 2003 to 2023 from the Web of Science Core Collection database. A total of 2044 articles were analyzed using VOSviewer, CiteSpace, and R-bibliometrix to examine publication trends, influential countries, institutions, authors, and journals, as well as emerging research topics.

**Results:**

Our analysis revealed a steady global increase in annual publications. The United States and China emerged as leading contributors, demonstrating substantial collaboration and investment in AMD research. Among institutions, the National Eye Institute in the United States and the University of Eastern Finland stood out for their prolific contributions. Key researchers such as Kaarniranta have been central to advancing the field, offering potential collaboration opportunities. Investigative Ophthalmology and Visual Science and Experimental Eye Research were identified as the top journals publishing significant work on inflammation in AMD. Keywords such as “oxidative stress,” “complement” “autophagy,” “macrophage,” and “microglia” were frequently mentioned, highlighting these as current research hotspots.

**Conclusion:**

This analysis underscores the importance of inflammation in AMD and maps the evolution of research, suggesting future directions that may offer new therapeutic strategies.

## 1. Introduction

Age-related macular degeneration (AMD) stands as a principal cause of irreversible visual impairment in elderly individuals world-wide. With the global aging population, the incidence of AMD is rising steadily, and it’s projected that by 2040, nearly 288 million individuals will suffer from this condition.^[1]^ This ocular pathology predominantly targets the macula, the central segment of the retina, which is indispensable for acute central vision. Two primary forms of AMD have been identified: dry (non-neovascular) and wet (neovascular) AMD. However, there are currently limited treatments for wet AMD and no FDA-approved therapy for dry AMD.^[2,3]^ As the world’s population ages, the repercussions of AMD become increasingly pertinent, driving the importance of innovative research and intervention approaches. While the exact etiology of AMD remains multifaceted and not fully elucidated, emerging evidence suggests that inflammation plays a pivotal role in its onset and progression.^[4]^ Inflammation is a cellular reaction to disturbances in cellular homeostasis, triggered by receptors sensing harmful patterns. It involves the release of cytokines and chemokines, attracting leukocytes to affected areas. While essential for defense, prolonged inflammation contributes to many chronic age-related diseases, including AMD.^[5]^

In the span of the past 2 decades, the nexus between inflammation and AMD has been a primary focus of global research. Investigations have spanned from oxidative stress to innate immunity, particularly emphasizing the complement system and innate immune cells in AMD’s pathophysiology.^[6-8]^ The symbiotic relationship between oxidative stress and inflammation, for instance, has garnered particular attention.^[9]^ Pathological oxidative insults have been shown to be detrimental, imparting damage to proteins, lipids, and DNA within retinal pigment epithelium (RPE) cells. Such perturbations potentiate pro-inflammatory cascades and foster macrophage recruitment. As a consequence, the inflammatory response, rather than being quiescent and homeostatic, escalates to a chronic state in the context of AMD.^[8,10]^ Simultaneously, the immunological facets of AMD’s etiopathogenesis have been under rigorous investigation.^[6]^ Noteworthy are the activated products of the complement system and intrinsic complement proteins have been found in AMD patients.^[11,12]^ Genetic endeavors have unveiled an array of risk alleles intimately associated with the complement pathway,^[13,14]^ further cementing the ties between AMD and innate immunity. Among these, the complement factor H (CFH) Y402H (Tyr402His) polymorphism stands out as a paramount genetic risk determinant for AMD.^[15-19]^ In addition, macrophages and microglia, pivotal constituents of innate immunity, have been observed to exhibit aberrant behavior and functionality under AMD conditions, potentially exacerbating retinal degeneration via inflammatory pathways.^[4,20,21]^ Albeit an extensive array of studies has delved into diverse facets of inflammation and its role in AMD, a holistic bibliometric analysis that paints a clear picture of the ongoing research trends and gaps in this domain remains scarce.

To address this gap, our current endeavor seeks to harness bibliometric methodologies to underscore the features of publications centered on inflammation in AMD, emphasizing prevailing research areas and concentration points. It is our aspiration that this work furnishes researchers in the AMD realm with a holistic perspective on the current landscape, aiding in pinpointing both contemporary challenges and potential future research trajectories. Ultimately, understanding the nexus between AMD and inflammation could pave the way for innovative therapeutic strategies, potentially reducing the global burden of this debilitating disease.

## 2. Materials and methods

### 2.1. Data collection

The Web of Science served as the primary source for identifying publications pertinent to this study. The search was refined to the “topic” scope, encompassing titles, abstracts, keywords, and additional relevant sections of the articles. The search strategy was formulated as follows: (((((TS= (“age related macular degeneration”)) OR TS= (“age related maculopathy”)) OR TS= (“age related maculopathies”)) OR TS= (“age related macular dystrophy”)) OR TS= (“age related macular dystrophies”)) AND TS= (*inflammation). This initial query yielded 5329 results. Constraints were applied to filter the Web of Science Core Collection (WoSCC) dataset by language (English only) and publication period (2003–2023), with the search executed on September 5th, 2023. Subsequently, a preliminary screening was conducted, followed by a meticulous review by 2 researchers, who scrutinized the titles, abstracts, and full texts of the retrieved documents. Any publication not explicitly addressing inflammation in AMD was excluded. Ultimately, 2044 manuscripts were deemed relevant and selected for in-depth analysis. The selection process is depicted in Figure S1, Supplemental Digital Content, https://links.lww.com/MD/R549.

### 2.2. Data analysis

VOSviewer version 1.6.18 was one of the primary tools employed. Prior to the analysis, data was standardized to ensure consistency. To minimize bias, author keywords were manually consolidated; for instance, variations such as “age related macular degeneration” and “age-related macular degeneration” were uniformly categorized under “AMD.” Regarding geographic information, Taiwan was included under the designation of China for analytical purposes. VOSviewer adeptly generated maps illustrating co-occurrence, co-citation, and co-authorship within the dataset. Typically, the size of the nodes or words corresponds to their frequency of occurrence, with larger nodes denoting higher frequency. The lines connecting 2 nodes symbolize their collaborative relationship, with thicker lines indicating more extensive collaboration. Additionally, node colors were assigned to reflect the average publication year of the associated documents.

CiteSpace 6.1. R2 serves as is another bibliometric software employed in this study, designed for generating visual maps derived from database-sourced data. It facilitated the creation of various knowledge-driven visual representations, including maps showcasing citation bursts, timeline views, and dual-map overlays. The configuration for these analyses was carefully set, with the parameters being: 1 year per slice for temporal analysis, application of minimal spanning tree and pruning sliced nets for data refinement, and an inclusion criterion of the top N = 40 most cited or occurring items, with all other settings retained at their default values. In the visualization maps detailing the co-occurrence of countries/regions and institutions, the nodes transition in color from blue to red to denote the progression of publication years from 2003 through to 2023, providing a dynamic overview of the research landscape over time.

Additionally, an online platform (https://bibliometric.com/app_v0) was utilized for conducting comparative analyses on the annual publication output among the top 10 contributing countries. For the graphical representation of annual publications, GraphPad Prism 8 and Microsoft Office Excel 365 were employed to craft a column chart and perform descriptive analyses, respectively. The impact factor (IF) scores referenced in this study were obtained from the 2022 Journal Citation Reports, ensuring the most current and relevant data was used for evaluation.

### 2.3. Ethical approval

This study is a bibliometric analysis based on data extracted from the Web of Science Core Collection database. Informed consent was therefore not applicable to this study.

## 3. Results

### 3.1. Publication outputs and trends

**Figure 1.**
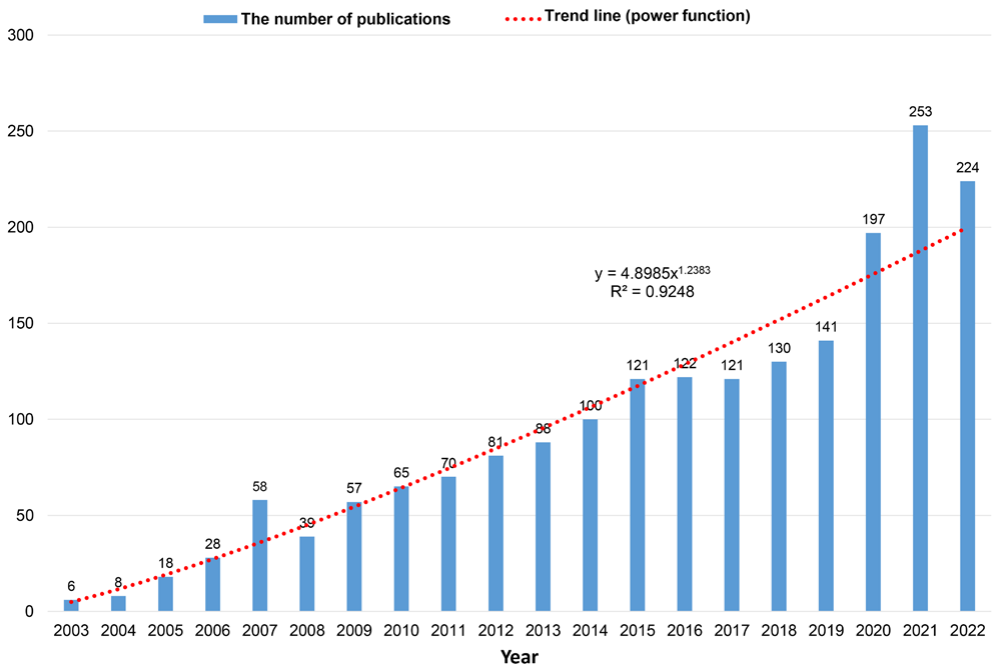
The annual publication trends on inflammation in AMD from 2003 to 2023. The bar chart shows the number of publications per year. The fitted power function curve (*y* = 4.8985 × ^1.2383^, *R*^2^ = 0.9248) demonstrates a strong upward trend in research output over time. AMD = age-related macular degeneration.

Employing the defined search criteria, we identified a total of 2044 papers focused on inflammation in AMD. The annual distribution of publications, as depicted in Figure 1, demonstrates a significant upward trajectory in research output from 2003 to 2023, surpassing the 100-publication mark for the first time in 2014. To quantitatively assess this growth pattern, a power function model (*y* = 4.8985 × ^1.2383^, *R*^2^ = 0.9248) was constructed, with *X* representing the year and *Y* the total number of publications per annum, highlighting a robust increase in scholarly interest over the observed period.

### 3.2. Most prolific countries/regions, funding agencies and institutions

**Figure 2.**
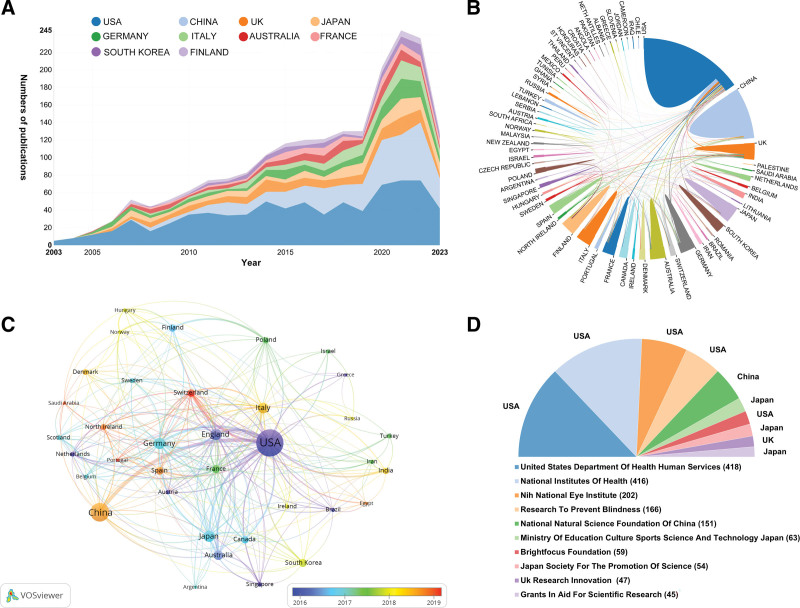
Distribution and collaboration of countries/regions and funding agencies. (A) Annual publication counts from the top 10 most productive countries. (B) Global collaboration network between countries. Lines connecting nodes (countries) represent collaborative relationships; thicker lines indicate stronger collaboration. (C) Co-authorship network among countries/regions. Node size corresponds to the total number of publications from that country. The color of nodes represents the average publication year (blue: earlier, red: more recent). Lines between nodes indicate co-authorship ties, with thicker lines denoting stronger collaboration. (D) The top 10 most active funding agencies and their respective countries. The number in parentheses indicates the count of supported publications. UK = United Kingdom, USA = United State of America.

A total of 2164 institutions across 78 countries/regions have contributed to publications on inflammation in AMD. Table 1 showcases the 10 leading countries in this research domain. The publication output from 2003 to 2023 reveals the United States as the forefront contributor with 775 publications, followed by China with 344, United Kingdom with 159, Japan with 142, Germany with 141, England with 136, and Australia with 103 (Fig. 2A). Notably, the United States boasts a centrality score of 0.53, significantly surpassing other nations, indicative of its pivotal role in fostering international collaborations in AMD inflammation research. Here, lines connecting 2 countries represent collaborative efforts (Fig. 2B). Figure 2C further elaborates on these connections, focusing on countries/regions with at least 10 publications. Regarding funding, the top 10 agencies, half of which are American with the United States Department of Health and Human Services emerging as the predominant source of research financing (Fig. 2D).

**Table 1:** Top 10 countries with publications regarding inflammation in AMD.

Rank	Country	Count	Percentage	Total citations	Average citation	*H*-index	Total link strength
1	Unite States	775	37.92%	35,161	45.37	97	45
2	China	382	18.69%	7009	18.35	44	18
3	Unite Kingdom	159	7.78%	6994	43.99	41	44
4	Japan	142	6.95%	3751	26.42	39	26
5	Germany	141	6.90%	4690	33.26	40	33
6	Italy	119	5.82%	3428	28.81	36	29
7	Australia	103	5.04%	3638	35.32	37	35
8	France	75	3.67%	3406	45.41	33	45
9	South Korea	70	3.42%	1278	18.26	21	18
10	Finland	66	3.23%	3272	49.58	31	50

AMD = age-related macular degeneration.

### 3.3. Forefront institutions

**Figure 3.**
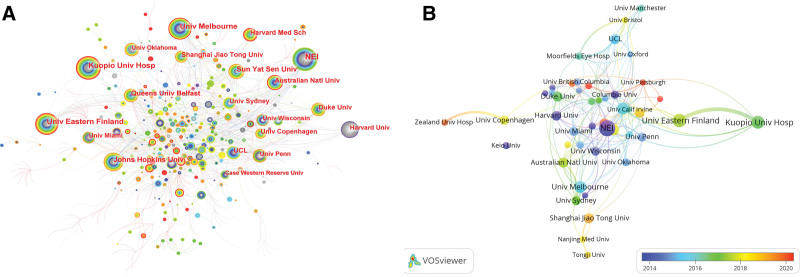
Institutional contributions and collaboration in inflammation in AMD research. (A) Network map of institutional co-occurrence. Node size represents the publication output of each institution. Lines between institutions indicate collaborative relationships, with thicker lines representing stronger collaboration. (B) Overlay visualization of institutional co-authorship. The color gradient of the nodes indicates the average publication year (blue: earlier, red: more recent), highlighting the temporal evolution of research activity. AMD = age-related macular degeneration.

Figure 3A illustrates the co-occurrences of institutions regarding researches on inflammation in AMD, with Table 2 listing the top 10 contributing institutions. The National Eye Institute (NEI) leads in publication volume (n = 72), closely followed by the University of Eastern Finland (n = 52), Kuopio University Hospital (n = 49), and the University of Melbourne (n = 44). Notably, the NEI boasts the highest count, underscoring its central role and collaborative impact in this field. Among these leading entities, 4 are based in the United States. Additionally, Figure 3B offers an insight into the co-authorship dynamics among these institutions, with a color gradient indicating the recency of average publication years − red denotes the most recent outputs.

**Table 2:** Top 10 institutions with publications regarding inflammation in AMD.

Rank	Institution	Count	Percentage	Total citation	Average citation	*H*-index	Total link strength	Country
1	National Eye Institute	72	3.52%	4276	59.39	37	36	Unite States
2	Univ Eastern Finland	52	2.54%	2364	45.46	27	57	Finland
3	Kuopio Univ Hosp	49	2.40%	2497	50.96	29	56	Finland
4	Univ Melbourne	44	2.15%	1482	33.68	23	34	Australia
5	Johns Hopkins Univ	43	2.10%	2345	54.53	33	30	Unite States
6	Sun Yat Sen Univ	40	1.96%	1455	36.38	20	11	China
7	Queens Univ Belfast	34	1.66%	1423	41.85	20	20	Unite Kingdom
8	Harvard Med Sch	33	1.61%	1800	54.55	29	14	Unite States
9	Shanghai Jiao Tong Univ	33	1.61%	626	18.97	16	7	China
10	Duke Univ	33	1.61%	458	13.88	22	10	Unite States

AMD = age-related macular degeneration.

### 3.4. Influential authors

**Figure 4.**
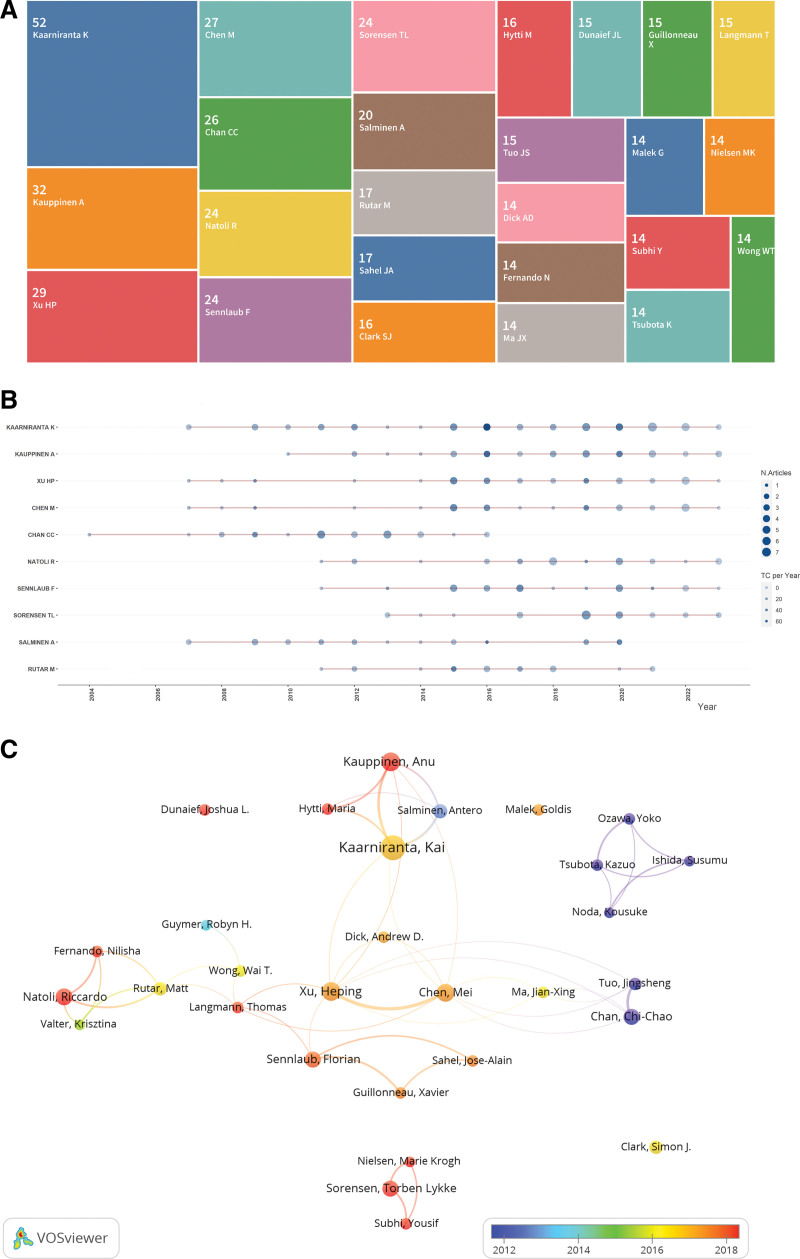
Analysis of influential authors. (A) Tree map of the top 25 most productive authors. The number within each box represents the author’s publication count. (B) Publication trends over time for the top 10 authors. The size of the data points corresponds to the number of publications in that year. (C) Collaboration network among the top 30 most prolific authors. Node size represents publication count. Lines between authors indicate co-authorship relationships.

Figure 4A showcases the 25 most influential authors in the study of inflammation in AMD, highlighting their publication output. The top 10 among these are further explored in Figure 4B and detailed in Table 3, illustrating their annual scholarly contributions. Kaarniranta, stands out as the leading author with an *H*-index of 27 and an impressive average citation rate of 51.55 per publication, underscoring the breadth and depth of their impact. Despite a lesser volume of work, Klein, R emerges as the most co-cited author, highlighting the profound influence of their contributions on the field (Table 4). Figure 4C maps the collaborative networks among the top 30 authors, with each author processing a minimum of 12 publications, revealing significant academic collaborations particularly among Kaarniranta, Kauppinen, Hatty and Salminen, as well as collaborations between Xu and Chen. This analysis not only highlights the productivity and scholarly impact of these authors but also the intricate web of collaborations that propel the advancement of research on inflammation in AMD.

**Table 3:** The top 10 most productive authors in study of inflammation in AMD.

Rank	Author	Count	Percentage	Total citation	Average citation		*H*-index	Total link strength	Country
1	Kaarniranta, K	47	2.30%	2423		51.55	27	34	Finland
2	Kauppinen, A	28	1.37%	1319		47.11	17	28	Finland
3	Xu, HePing	28	1.37%	1656		59.14	17	28	Unite States
4	Chen, Mei	26	1.27%	1606		61.77	16	26	Unite Kingdom
5	Natoli, R	24	1.17%	743		30.96	21	19	Australia
6	Chan, Chi-Chao	23	1.13%	1585		68.91	15	15	Unite States
7	Sennlaub, F	23	1.13%	1085		47.17	17	16	France
8	Sorensen, T L	22	1.08%	370		16.82	12	14	Denmark
9	Salminen, A	18	0.88%	1550		86.11	18	18	Finland
10	Rutar, M	17	0.83%	911		53.59	12	16	Australia

AMD = age-related macular degeneration.

**Table 4:** The top 10 co-cited authors in study of inflammation in AMD.

Rank	Co-cited author	Citation frequency	Total link strength	*H*-index		Country
1	Klein	830	673	103	Unite States	
2	Seddon	673	592	98	Unite States	
3	Hageman	628	593	51	Unite States	
4	Anderson	547	526	44	Unite States	
5	Ambati	473	445	43	Unite States	
6	Curcio	374	327	78	Unite States	
7	Jonas	361	239	156	Switzerland	
8	Chen	356	320	41	Unite Kingdom	
9	Penfold	335	310	32	Australia	
10	Johnson	331	319	22	Unite States	

AMD = age-related macular degeneration.

**Figure 5.**
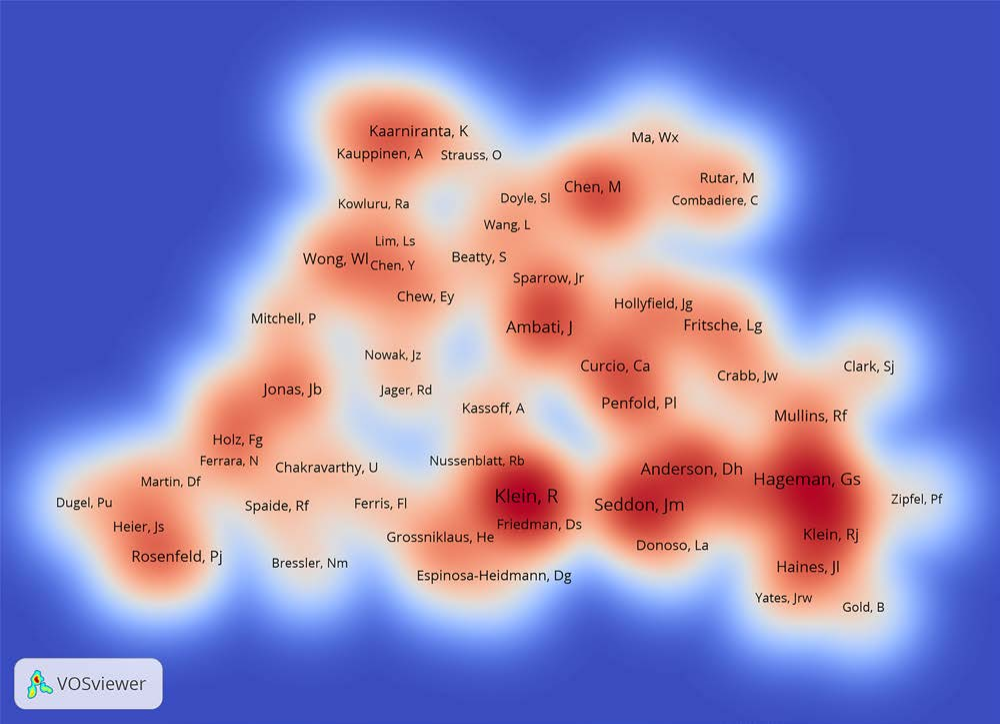
Density visualization of co-cited authors. The size of each label corresponds to the co-citation frequency of the author. Larger labels indicate higher co-citation counts.

Figure 5 unveils the co-citation landscape of authors, zooming in on those who have amassed 110 or more co-citations. Topping the chart, Klein emerges with a remarkable 830 co-citations, closely followed by Seddon with 673 and Hageman with 628 co-citations. Details of these and other top 10 co-cited authors, along with their co-citation figures, are meticulously listed in Table 4. This elite group of authors commands profound respect and recognition across the research domain, as reflected in their substantial co-citation tallies. Through the lens of prolific and co-cited authors, this analysis illuminates their scholarly output, pivotal roles, and the network of collaborations that underscore the collective pursuit of knowledge in the field of inflammation in AMD.

### 3.5. Principal journals

**Figure 6.**
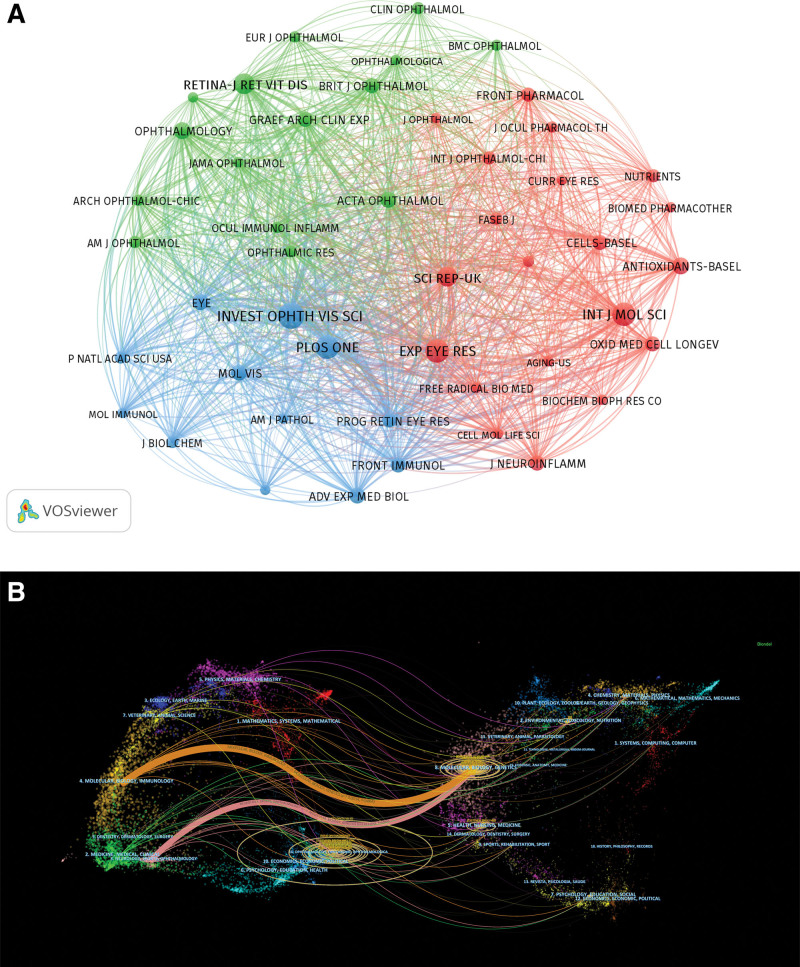
Journal analysis. (A) Co-citation network map of journals. Node size represents the number of publications (for citing journals) or citation frequency (for cited journals). (B) Dual-map overlay of journals. The left cluster represents citing journals, and the right cluster represents cited journals. The colored curves trace the citation pathways between journal groups, indicating that research published in Molecular/Biology/Genetics and Medicine/Medical/Clinical journals cites work from Molecular/Biology/Immunology and Neurology/Sports/Ophthalmology journals.

The analysis encompassed articles from 529 journals, with 47 journals each publishing at least 10 papers, as illustrated in Figure 6A. Table 5 lists the top 10 journals contributing to the field, led by Investigative Ophthalmology and Visual Science with 90 publications, followed by Plos One with 70 publications, Experimental Eye Research with 68 publications, International Journal of Molecular Sciences with 66 publications, and Retina-The Journal of Retinal and Vitreous Diseases with 51 publications. Significantly, all journals in the top 10 have an IF above 2 for the year 2022, with 4 achieving Q1 category according to the journal citation reports division. Besides, Table 6 presents the top 10 co-cited journals within this field. Investigative Ophthalmology and Visual Science emerged as the most frequently cited journal, accumulating 10,690 citations, followed by Ophthalmology with 5063 citations, and Proceedings of the National Academy of Sciences of the United States of America with 3596 citations. Notably, the average IF for these top 10 journals is 7.2. Among them, 3 journals boast IFs >10, and 7 are classified within the Q1 category, indicating a strong preference for citing from journals recognized for their high quality. To visualize the interconnectedness of these journals, a dual-map overlay was created using CiteSpace, shown in Figure 6B. This overlay revealed 2 primary citation trajectories, indicating that works published in journals categorized under Molecular/Biology/Immunology or Neurology/Sports/Ophthalmology are predominantly cited by articles in Molecular/Biology/Genetics.

**Table 5:** **The top 10 productive journals in research of inflammation in AM**D.

Rank	Journal	Count	Percentage	Average citation	IF 2022	JCR quartile 2022
1	Invest Ophth Vis Sci	90	4.40%	36.39	4.4	Q1
2	Plos One	70	3.42%	30.14	3.7	Q2
3	Exp Eye Res	68	3.33%	24.16	3.4	Q2
4	Int J Mol Sci	66	3.23%	13.06	5.6	Q1
5	Retina-J Ret Vit Dis	51	2.50%	30.06	3.3	Q2
6	Sci Rep-Unite Kingdom	45	2.20%	24.24	4.6	Q2
7	Ophthalmology	33	1.61%	56.58	13.7	Q1
8	Antioxidants-Basel	32	1.57%	11.47	7	Q1
9	Mol Vis	30	1.47%	25.93	2.2	Q3
10	Adv Exp Med Biol	30	1.47%	15.90	3.65 (2021)	Q2

AMD = age-related macular degeneration.

**Table 6:** The top 10 co-cited journals in research of inflammation in AMD.

Rank	Co-cited journal	Citation frequency	Total link strength	IF 2022	JCR quartile 2022
1	Invest Ophth Vis Sci	10,690	6,77,142	4.4	Q1
2	Ophthalmology	5063	2,98,809	13.7	Q1
3	P Natl Acad Sci	3596	2,36,461	11.1	Q1
4	Arch Ophthalmol-Chic	3589	2,39,432	4.399	Q1
5	Exp Eye Res	3327	2,30,275	3.4	Q2
6	Plos One	2998	1,97,542	3.7	Q2
7	Am J Ophthalmol	2945	2,02,910	4.2	Q1
8	J Biol Chem	2890	1,92,283	4.8	Q2
9	Prog Retin Eye Res	2733	1,81,945	17.8	Q1
10	Brit J Ophthalmol	2193	1,57,539	4.1	Q1

AMD = age-related macular degeneration, IF = impact factor, JCR = Journal Citation Report.

### 3.6. Co-cited references and citation burst

**Figure 7.**
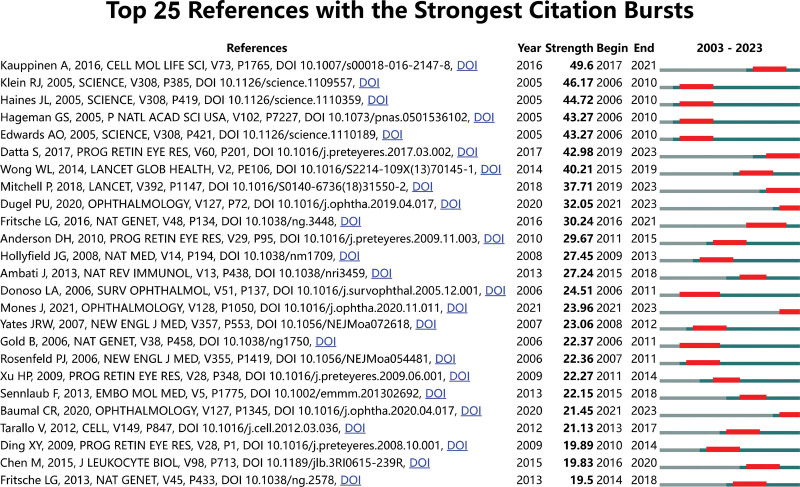
Top 25 references with the strongest citation bursts. The red bars on the timeline indicate the period during which each reference experienced a significant surge in citations. The strength value quantifies the intensity of each citation burst.

In the Table S1, Supplemental Digital Content, https://links.lww.com/MD/R549, we have compiled a list detailing the top 10 most cited papers. Notably, the 10 highest cited articles among these have each achieved over 480 citations. Furthermore, Table 7 outlines the top 10 references with the highest co-citation frequency, with 3 of these studies garnering more than 300 co-citations each. Additionally, we performed an analysis of citation bursts with CiteSpace to pinpoint papers experiencing significant spikes in citations over the past 20 years (Fig. 7). Remarkably, the article authored by Kauppinen in 2016 stands out as the most cited and also ranks highly in terms of co-citation within the AMD inflammation research domain, boasting the most substantial citation burst (strength = 49.6) and drawing considerable focus from the academic community between 2017 and 2021.

**Table 7:** The top 10 most co-cited references regarding inflammation in AMD.

Rank	Title (publication year)	First author	Journal	Co-citation
1	Complement factor H polymorphism in age-related macular degeneration (2005)	Klein	Science	324
2	Complement factor H polymorphism and age-related macular degeneration (2005)	Edwards	Science	304
3	Global prevalence of age-related macular degeneration and disease burden projection for 2020 and 2040: a systematic review and meta-analysis (2014)	Wong	Lancet Glob Health	304
4	Complement factor H variant increases the risk of age-related macular degeneration (2005)	Haines	Science	296
5	A common haplotype in the complement regulatory gene factor H (HF1/CFH) predisposes individuals to age-related macular degeneration (2005)	Hageman	P Natl Acad Sci Unite States	282
6	Ranibizumab for neovascular age-related macular degeneration (2006)	Rosenfeld	New Engl J Med	184
7	Inflammation and its role in age-related macular degeneration (2016)	Kauppinen	Cell Mol Life Sci	173
8	Oxidative damage-induced inflammation initiates age-related macular degeneration (2008)	Hollyfield	Nat Med	164
9	The pivotal role of the complement system in aging and age-related macular degeneration: Hypothesis re-visited (2010)	Anderson	Prog Retin Eye Res	149
10	Immunology of age-related macular degeneration (2013)	Ambati	Nat Rev Immunol	147

AMD = age-related macular degeneration.

### 3.7. Subject categories

**Figure 8.**
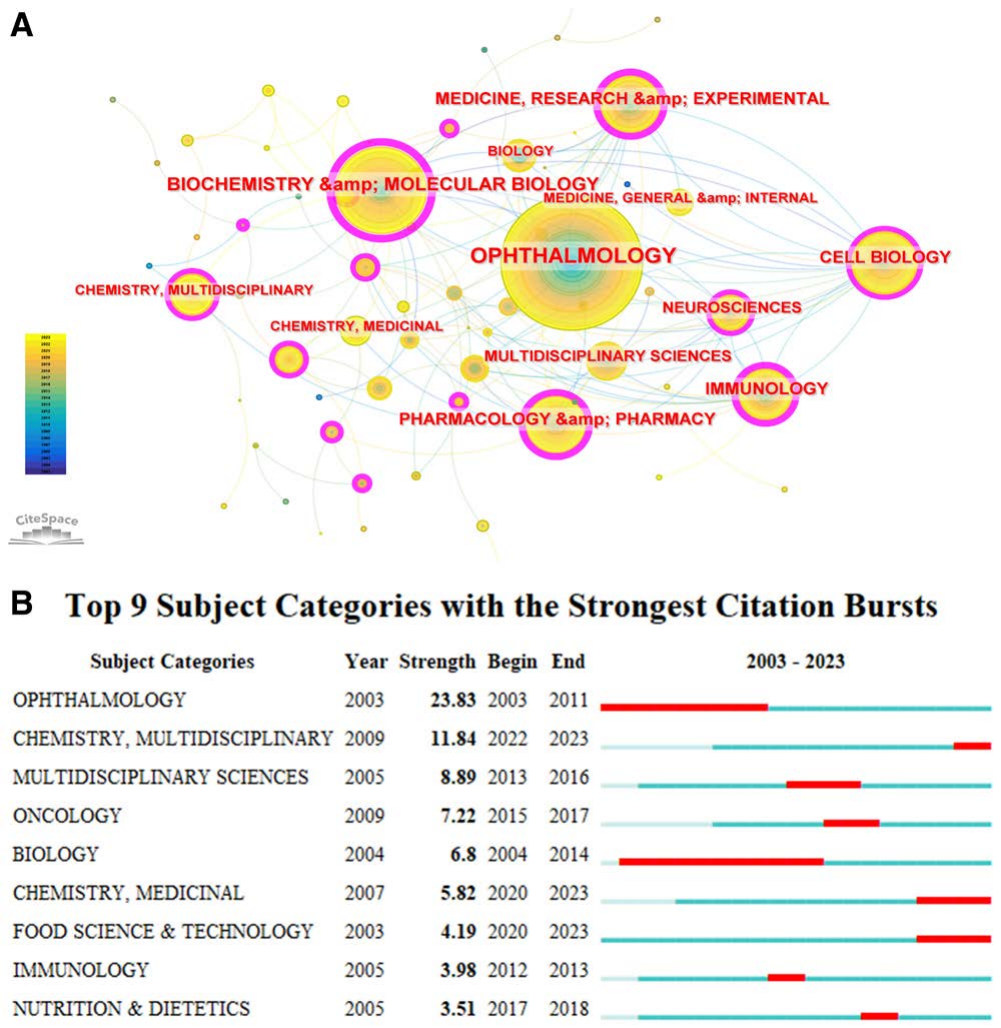
Analysis of subject categories. (A) Co-occurrence network of subject categories. Node size represents the frequency of the category. (B) Top 9 subject categories with the strongest citation bursts. The red bars indicate the time period of the burst.

The co-occurrence network map visualizes the subject categories appearing at least 50 times, as shown in Figure 8A. Ophthalmology is the most frequent category with 737 occurrences, followed by Biochemistry and Molecular Biology (344 occurrences), Cell Biology (227), and Medicine, Research and Experimental (200). These categories are prominent in AMD research, reflecting their significant contributions to the field. Notably, Biochemistry and Molecular Biology exhibits the highest betweenness centrality at 0.43, underscoring its pivotal role in bridging interdisciplinary research, followed by Pharmacology and Pharmacy (0.31), Medicine, Research and Experimental (0.29), and Immunology (0.23; Table 8). Furthermore, a citation burst analysis was conducted to identify subject categories that have shown notable increases in citations over the past 2 decades. The top 9 categories with the most intense bursts were determined by setting the burst duration to 1 year, as detailed in Figure 8B. Ophthalmology exhibited the most significant citation burst with a strength of 23.83, capturing substantial academic interest from 2003 to 2011. Other categories such as Chemistry, Medicinal, Food Science & Technology, and Chemistry, Multidisciplinary, which continue to be active through 2023, signify the leading-edge disciplines in inflammation research within AMD.

**Table 8:** The top 10 subject categories in frequency and betweenness centrality.

Rank	Subject categories	Count	Rank	Subject categories	Centrality
1	Ophthalmology	737	1	Biochemistry And Molecular Biology	0.43
2	Biochemistry and Molecular Biology	344	2	Pharmacology and Pharmacy	0.31
3	Cell Biology	227	3	Medicine, Research and Experimental	0.29
4	Medicine, Research and Experimental	200	4	Immunology	0.23
5	Pharmacology and Pharmacy	169	5	Cell Biology	0.19
6	Immunology	158	6	Biotechnology and Applied Microbiology	0.19
7	Multidisciplinary Sciences	148	7	Nanoscience and Nanotechnology	0.18
8	Neurosciences	107	8	Chemistry, Multidisciplinary	0.17
9	Chemistry, Multidisciplinary	86	9	Engineering, Biomedical	0.16
10	Biology	62	10	Chemistry, Applied	0.15

### 3.8. Co-occurring author keywords

**Figure 9.**
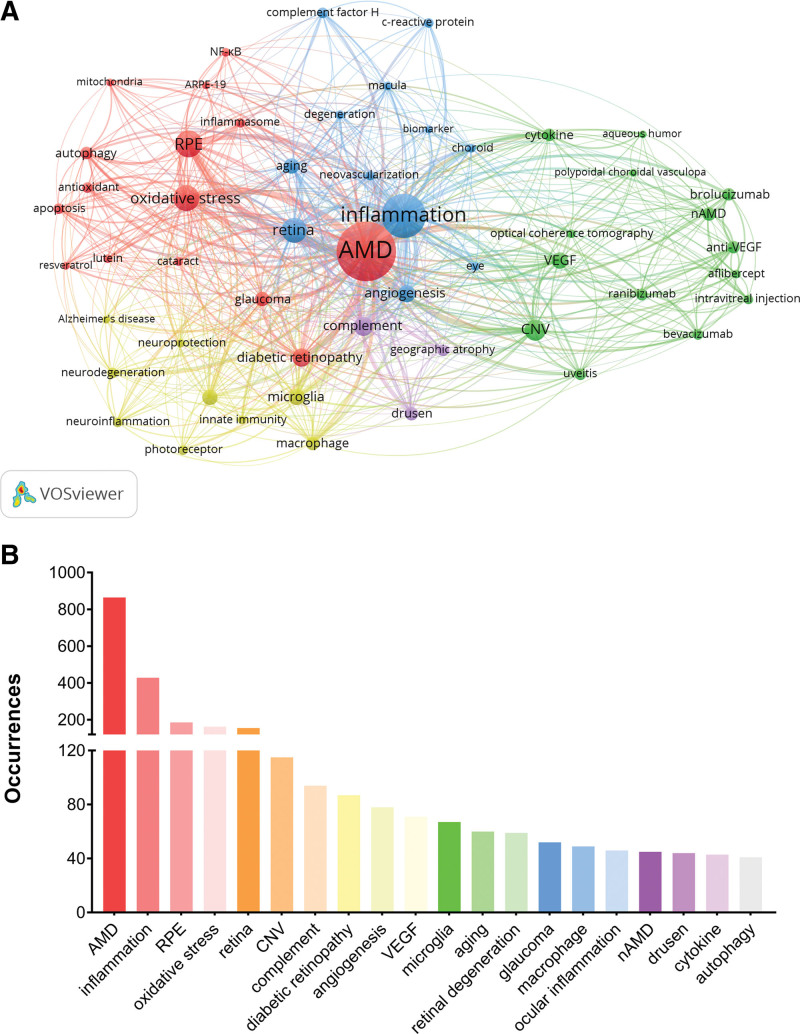
Author keywords analysis. (A) Overlay visualization of co-occurring author keywords. Node size represents the frequency of the keyword. The color of the nodes indicates the average publication year (blue: earlier, red: more recent). (B) The 20 most frequently used author keywords. AMD = age related macular degeneration, CNV = choroidal neovascularization, nAMD = neovascular age related macular degeneration, RPE = retinal pigment epithelium, VEGF = vascular endothelial growth factor.

In our study, we utilized author keywords to delve deeply into the prevalent research themes and evolving trends within the domain of inflammation in AMD. Among 3229 identified author keywords, we focused on 53 that appeared no <15 times, leading to the overlay visualization depicted in Figure 9A. This map underscores the intricate connections and relationships among these keywords, showcasing the focal points and areas of keen interest within the inflammation in AMD research domain. Furthermore, Figure 9B lists the top 20 author keywords by frequency, highlighting prominent terms such as “AMD,” “inflammation,” “RPE,” “oxidative stress,” “retina,” “choroidal neovascularization (CNV),” and “complement.” These keywords stand out as the most significant and recurrently mentioned, indicating their central role in current research discussions.

**Figure 10.**
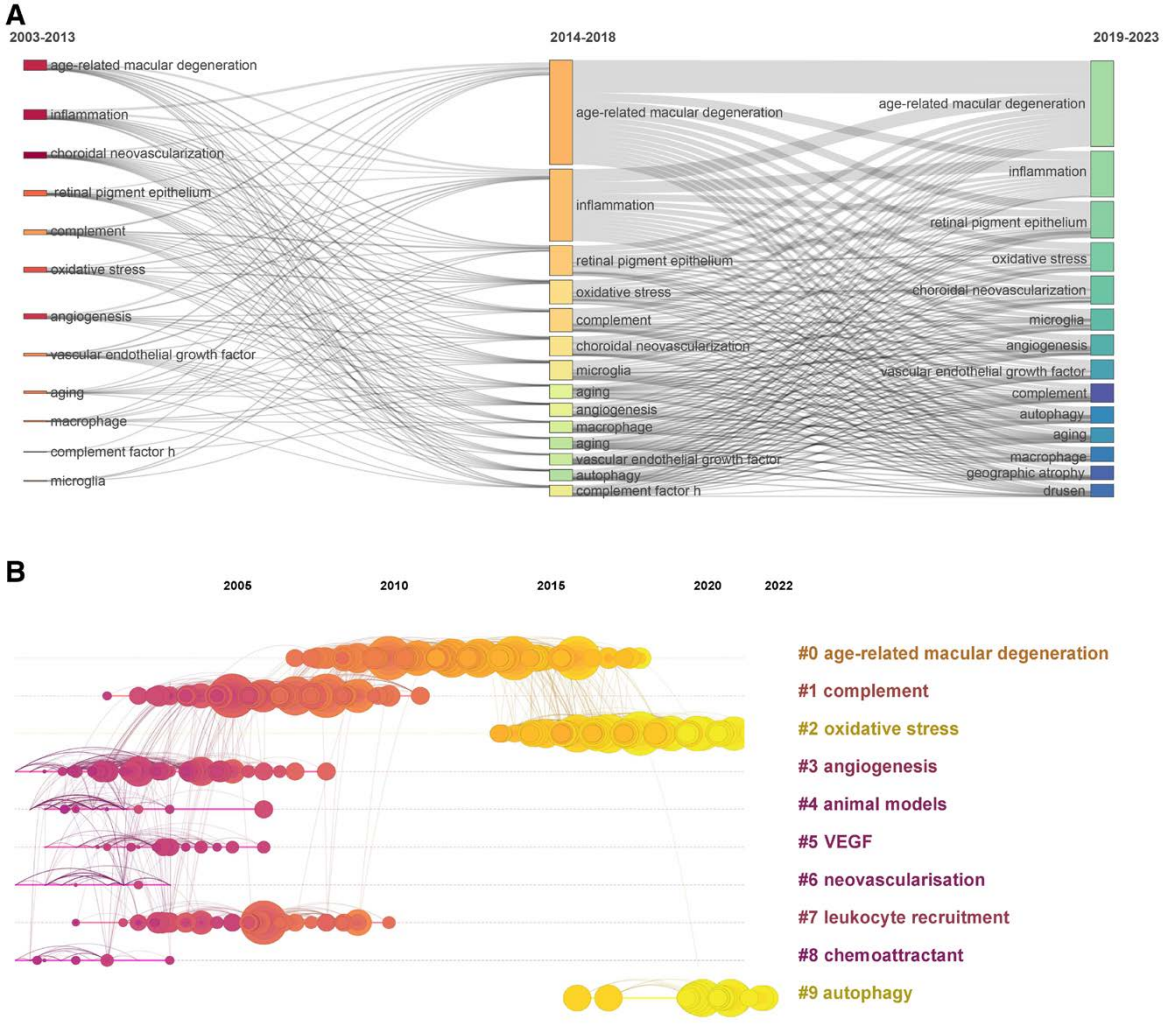
Thematic evolution analysis. (A) Three-field plot showing the evolution of the top author keywords across 3 time periods. The width of the bands connecting the fields is proportional to the co-occurrence frequency of keywords between periods. (B) Timeline view of co-cited references clustered by research theme. The clusters (right) represent major thematic areas. The timespan of each cluster shows its period of activity. VEGF = vascular endothelial growth factor.

Moreover, Figure 10A presents a thematic evolution analysis, pinpointing the top 15 author keywords based on their prominence within specific timeframes. This analysis employs a 3-field plot to trace the progression of inflammation in AMD research across 3 separate periods. Notably, our findings underscore the heightened focus on “oxidative stress,” “microglia,” and “autophagy” within the last 5 years. These subjects have ascended as central themes, reflecting their escalating significance and pertinence in recent explorations within the realm of AMD research.

Figure 10B showcases a timeline-view graph featuring nodes that represent co-cited references, systematically categorized into 18 distinct thematic clusters through an analysis of author keywords (modularity *Q* value = 0.7746, weighted mean silhouette value = 0.9147). These clusters include: “age-related macular degeneration” (#0), “complement ‘(#1), ‘oxidative stress’ (#2),’ angiogenesis” (#3), “animal models” (#4), “VEGF” (#5), “neovascularisation” (#6),”leukocyte recruitment” (#7), “ chemoattractant “ (#8), and “autophagy” (#9). Remarkably, “oxidative stress “and “ autophagy “ represent 2 emerging research directions within the field of inflammation in AMD in recent years. These novel areas of investigation signify the evolving research landscape, reflecting the scientific community’s growing interest in the aspect of autophagy and oxidative stress for exploring the pathogenesis of AMD.

**Figure 11.**
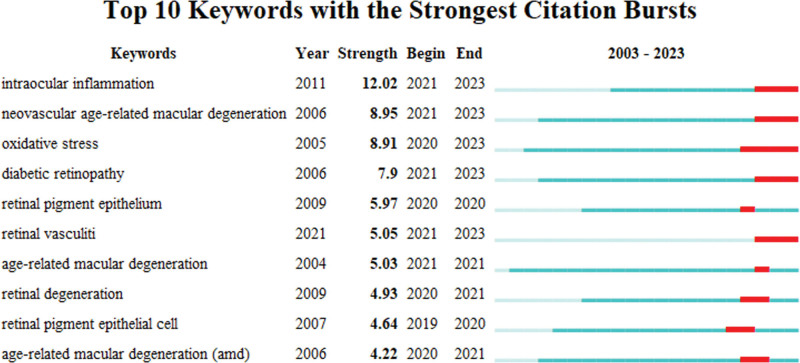
Top 10 author keywords with the strongest citation bursts. The red bars highlight the time period during which each keyword experienced a significant increase in citations, indicating emerging research trends.

Additionally, Figure 11 displays the top 10 author keywords with the most significant citation bursts, highlighting “intraocular inflammation,” “neovascular age-related macular degeneration,” “oxidative stress,” and “diabetic retinopathy” as subjects of significant interest in recent 3 years. These keywords underscore the current research directions, capturing the focal areas of heightened scholarly engagement. The clustering approach used in this analysis, with a modularity *Q* of 0.68 and a weighted mean silhouette value of 0.89, affirms the method’s logical structure and efficacy in categorizing and pinpointing significant thematic clusters derived from citation trends.

Taken together, this bibliometric analysis demonstrates that research on inflammation in AMD has gradually shifted from descriptive pathological observations toward mechanistic and cellular–molecular investigations. While oxidative stress, complement dysregulation, and innate immune cell activation − particularly involving macrophages and microglia − remain the core mechanistic pillars, recent trends indicate growing interest in autophagy dysfunction, mitochondrial metabolic stress, and lipid-mediated inflammatory signaling. However, current studies largely examine these pathways in isolation, and an integrated perspective explaining how these mechanisms interact to perpetuate chronic retinal inflammation is still lacking. Future research should therefore prioritize cross-omics integration, high-resolution immune microenvironment mapping, and therapeutic strategies aimed at restoring immune-metabolic homeostasis rather than solely suppressing single inflammatory mediators.

## 4. Discussion

### 4.1. Overview

The growing research fronts and future research interests in the field of inflammation in AMD over the past nearly 20 years are identified in this study. The data extracted from WoSCC database showed that 9089 authors from 2164 institutions in 78 countries/regions published 2044 papers related to inflammation in AMD in 527 journals between January 1, 2003 and September 5, 2022.

The analysis of publication outputs and trends concerning inflammation in AMD (Fig. 1) revealed a significant increase in scholarly interest and research efforts in this area, particularly noticeable from the year 2014 onwards, where for the first time, annual publications exceeded the 100-paper mark. This pivotal increase can be attributed to a paradigm shift in understanding AMD’s pathophysiology, emphasizing inflammation’s role.^[14]^ This period also saw significant technological advancements in medical research, increased focus on AMD due to its rising prevalence in an aging population, and enhanced interdisciplinary collaboration.^[1]^ These factors collectively spurred a marked increase in research activities, as reflected in the exponential growth in the number of publications, aligning with the observed trend in the power function model.

### 4.2. Analysis of geographical and institutional contributions

The geographic and institutional contributions to inflammation in AMD research, as illustrated in our analyses, prominently feature the United States and China as the leading contributors, with 1.3-fold more publications than the combined all of the next top 10 countries. The United States emerges as a clear leader in this domain, contributing the highest number of publications. This leadership position likely results from a combination of robust funding mechanisms, evidenced by the most significant financial support for research and a strong emphasis on medical research and development, evidenced by its central role in the international collaboration network.

Analogous to the geographic distribution, the leading 10 institutions with the highest publication output are primarily located in the United States. National Eye Institution, Johns Hopkins University, Harvard Medical School and the Duke University, the best scientific research institutions in the United States, occupy 4 of the top 10 slots in the list of most prolific institutions. The NEI emerges as a prominent leader in this realm, with the highest publication count. Its leading position, coupled with the highest centrality score of 0.13, not only underscores its prolific output but also its central role in fostering collaborative research efforts. The NEI’s influence in the field is indicative of its commitment to advancing our understanding of AMD, especially in relation to inflammation. Furthermore, the active international cooperation between various institutions underscores the significance of collaboration in fostering growth within this field.

### 4.3. Analysis of leading researchers and their collaborative networks

Identifying influential scholars in a particular field provides early-career researchers with insightful guidance and direction. In the data presented in Figure 4 and enumerated in Table 3, we highlighted the authors with the highest number of publications. Kaarniranta from Department of Ophthalmology, Institute of Clinical Medicine, and University of Eastern Finland leads the list with 52 articles and an average citation count of 51.55 per paper. The work of Kaarniranta in the field of AMD represents a significant contribution to our understanding of the disease’s pathogenesis, particularly in relation to the role of inflammation. Kaarniranta’s research delves deeply into the complex mechanisms at play in AMD, emphasizing the critical role of oxidative stress, mitochondrial function, angiogenesis and autophagy, and their interplay with inflammation in the degeneration of the RPE, which opens up new pathways for research and potential therapeutic strategies for AMD.^[22-25]^

Kauppinen, Anu as the second most prolific author in this field, also made substantial contribution to unraveling the intricacies of inflammatory processes in AMD. Kauppinen’s work, often in collaboration with Kaarniranta, has been instrumental in deepening the understanding of how inflammation interacts with other cellular mechanisms, such as oxidative stress and autophagy, in the context of AMD.^[5,25-30]^ This collaboration likely facilitates a more comprehensive exploration of AMD pathogenesis, considering various interconnected pathways that contribute to the disease.

As for the co-cited authors shown in Figure 5 and Table 4, Klein, Ronald from the Department of Ophthalmology and Visual Sciences, University of Wisconsin-Madison and Johanna Seddon from Department of Ophthalmology, Tufts University School of Medicine, have published several highly cited review papers on AMD.^[31-34]^ These review articles help other scholars quickly and accurately understand the field of pathogenesis of AMD. In short, the top 10 authors with the most influential publications and the most co-citations have been leading the entire discipline forward.

### 4.4. Analysis of journals leading the field

Journals are an important vector for the dissemination of academic research results. We summarized the co-citation visualization network of the most influential journals in the field of inflammation in AMD, making it easier for researchers to choose the most suitable journals to submit papers. As shown in Figure 6 and Table 5, Investigative Ophthalmology and Visual Science, Plos One, Experimental Eye Research, International Journal of Molecular Science and Retina-Journal of Retinal and Vitreous Diseases take up the top 5 positions.

Investigative Ophthalmology and Visual Science, leading with the highest number of publications in this domain, is renowned for its comprehensive coverage of all aspects of ophthalmology and visual science. This journal’s focus on cutting-edge research in ocular diseases, mechanisms of visual function, and sight preservation aligns seamlessly with the evolving landscape of AMD research.^[35,36]^ The substantial number of papers published in IOVS on inflammation in AMD highlights its importance as a central repository of high-impact research findings. It is a testament to the journal’s reputation and influence that researchers prioritize it for submissions of significant studies, which, in turn, propels advancements in understanding and potentially managing AMD.

On the other hand, Experimental Eye Research offers a unique blend of experimental and investigative studies focused on the eye and vision science. Its emphasis on the molecular, cellular, and physiological mechanisms underlying eye diseases makes it an invaluable resource for researchers exploring the complex interplay between inflammation and AMD.^[37]^ The journal’s commitment to publishing studies that elucidate the pathophysiological processes at play in AMD, including oxidative stress, mitochondrial dysfunction, and autophagy,^[37-39]^ provides critical insights that bridge basic science with potential therapeutic applications.

### 4.5. Analysis of key co-cited references

The analysis of the top co-cited references in the context of inflammation and AMD underscores the pivotal discoveries and critical shifts in the understanding of AMD’s pathogenesis. The leading 3 articles, as highlighted in Table 7, collectively emphasize the significant role of the complement system in AMD and the global disease burden.

Klein’s study published on Science, with the highest co-citation count, marks a cornerstone in AMD research by identifying the association between CFH polymorphism and AMD. The revelation that genetic variations in CFH, a regulator of the complement pathway, could significantly increase AMD risk highlighted the integral role of the immune system, particularly the complement cascade, in AMD pathogenesis.^[18]^ This discovery prompted a paradigm shift towards understanding AMD not just as a degenerative condition but as one with a strong inflammatory component.

Published concurrently with Klein’s study, Edwards’s work further cemented the genetic link between CFH polymorphism and AMD.^[16]^ The slightly lower co-citation count compared to Klein’s article does not diminish its importance; rather, it underscores the collaborative emergence of evidence pointing to the complement system’s dysfunction as a critical factor in AMD. These parallel findings have significantly influenced subsequent research directions, focusing on unraveling the mechanisms by which the complement system contributes to AMD.

The third most co-cited paper was a systematic review article by Wong WL. This publication shifts the focus from molecular mechanisms to the epidemiological aspect of AMD, providing a comprehensive analysis of its global prevalence. By projecting the disease burden into future decades, Wong’s study underscores the pressing need for targeted research and effective public health strategies to manage AMD, highlighting its status as a major global health concern.^[1]^ This work serves as a call to action for the research community to prioritize AMD due to its significant impact on the aging population.

### 4.6. Analysis of top references with the strongest citation burst

The analysis of citation bursts, as listed in Figure 7, provides insight into the articles that have significantly influenced the field of AMD research over a specific period. The leading 3 articles, as identified, mark pivotal shifts in our understanding of AMD, particularly emphasizing the roles of inflammation and genetic predispositions in its pathogenesis.

Kauppinen’s 2016 publication stands at the forefront with the strongest citation burst, indicating its profound impact on the field. This article synthesized existing knowledge and presented a compelling argument for the central role of inflammation in AMD.^[5]^ It meticulously outlined how chronic inflammation acted as both a consequence and a driver of AMD progression, bridging the gap between basic inflammatory mechanisms and clinical manifestations of the disease. By consolidating evidence on various inflammatory markers, pathways, and the interactions between innate and adaptive immunity in AMD, Kauppinen’s work has significantly shaped subsequent research directions, focusing on inflammation as a therapeutic target.

The second most cited burst reference is Klein’s seminal work in 2005 provided the first genetic link between CFH polymorphism and AMD, marking a breakthrough in our understanding of the disease’s genetic underpinnings.^[18]^ Published in the same year as Klein’s study, Haines’s article further solidified the link between CFH variants and AMD.^[17]^ The joint contributions of Klein and Haines have fundamentally altered our understanding of AMD, establishing a genetic and inflammatory basis for the disease. Their work has not only elucidated key aspects of AMD pathogenesis but also opened new avenues for research and treatment, emphasizing the importance of the complement system in AMD.

### 4.7. Current research focuses and predictions for future directions

The comprehensive analysis of author keywords and thematic evolution in the domain of inflammation in AMD, as illustrated in Figure 8, provides a panoramic view of the current hot topics and hints at future research trends within this critical area of study. Our findings underscore the dynamic nature of AMD research, highlighting the shift towards exploring intricate mechanisms like oxidative stress, leukocytes (macrophage and microglia) activity, and autophagy in recent years.

It is important to note that the research foci and mechanistic insights discussed below are inferred from bibliometric trends − such as keyword frequency, citation bursts, and thematic evolution − rather than derived from direct experimental evidence within this study. These trends reflect the collective focus of the research community and suggest promising avenues for future experimental validation

#### 4.7.1. Oxidative stress in AMD

The prominence of “oxidative stress” in our study’s keyword analysis highlights its integral role in AMD research, suggesting that the academic community has identified it as a crucial area of investigation. This is corroborated by the consistent appearance of oxidative stress across multiple highly co-cited and burst-analysis articles, indicating a strong and growing interest in understanding its specific contributions to AMD.

Oxidative stress, resulting from an imbalance between reactive oxygen species production and the antioxidative defense mechanism, has been extensively documented as a key contributor to RPE dysfunction − a crucial event in AMD pathobiology.^[40]^ The retina, particularly the RPE, is highly susceptible to oxidative damage due to its exposure to constant light, high metabolic activity, and the abundance of polyunsaturated fatty acids. Literature indicates that environmental factors, such as smoking and a high-fat diet, exacerbate oxidative stress, transitioning it from a physiological to a pathological role in AMD.^[41]^ Moreover, the disruption of cytoprotective pathways, including mitochondrial dynamics and the Nrf2 signaling system, further aggravated oxidative stress and inflammation, underpinning the degenerative changes observed in AMD.^[10]^ Intriguingly, recent studies have emphasized a positive feedback loop between oxidative stress and inflammation, suggesting that treatments aimed at mitigating reactive oxygen species and bolstering antioxidant defenses could offer promising avenues for AMD management.^[8,42]^

Future research in the realm of oxidative stress and AMD should concentrate on unraveling the complex mechanisms that link oxidative damage to the progression of AMD, particularly focusing on how oxidative stress interacts with inflammatory responses within the RPE. Additionally, the development and clinical evaluation of potent, retina-targeted antioxidants that can effectively counteract oxidative stress holds promise for slowing or preventing AMD progression. Exploring innovative strategies, such as energy supplementation, to enhance the retina’s intrinsic antioxidative and repair capabilities also emerges as a crucial research avenue.

#### 4.7.2. Complement in AMD

The analysis indicates a concerted research effort aimed at deciphering the role of complement activation in AMD, highlighting it as a key area of interest within the field. This attention is driven by the system’s intricate involvement in inflammatory responses and it’s potential as a therapeutic target, as evidenced by numerous clinical trials exploring complement inhibitors for AMD treatment.

The exploration of the complement system in AMD has yielded substantial insights, particularly in understanding its role in the disease’s progression and the development of therapeutic strategies. Genetic studies have illuminated the importance of the complement system in AMD pathogenesis, revealing specific risk alleles, including CFH, ARMS2/HTRA1, CFB, CFI, C2 and C3 variant alleles, and their associations with the disease. The discovery of the complement system’s dysregulation in AMD pathogenesis has opened new therapeutic avenues, particularly for geographic atrophy in dry AMD.^[43]^ Clinical trials investigating complement inhibitors have shown promise, with several candidates advancing to phase III trials after demonstrating efficacy in slowing the progression of geographic atrophy in phase II studies. This progress underscores the potential of targeting complement pathways as a strategy to mitigate AMD progression, especially for dry AMD, which currently lacks effective treatment options.^[44,45]^ Moreover, compelling evidence has highlighted the potential of integrating complement modulation with antiangiogenic treatments, offering new hope for neovascular AMD.^[46]^

There’s a pressing need to understand the functional consequences of identified genetic risk factors and their interactions, which may unveil new therapeutic targets. Additionally, exploring novel therapeutic strategies, such as complement gene therapy and combination therapies, represents a promising avenue for addressing the unmet needs in AMD treatment. The exploration of the complement system in AMD stands at a pivotal junction, with the potential to significantly advance our understanding and management of this complex, multifactorial disease.

#### 4.7.3. Macrophage and microglia activity in AMD

Our bibliometric analysis has highlighted the significance of macrophage and microglia activity within the scope of AMD research. This insight is drawn from a thorough examination of recurring keywords and thematic trends, underscoring the scientific community’s growing interest in the roles these immune cells play in AMD’s pathogenesis. The analysis suggests an investigative emphasis on how these cells mediate the balance between neuroprotection and neurodegeneration, underlining their dual role in retinal health and disease.

Macrophages, derived from circulating monocytes, and microglia, as resident macrophages, recruited to sites of retinal injury, are instrumental in maintaining retinal integrity through debris clearance. However, their dysfunction or maladaptive responses contribute significantly to AMD pathogenesis.^[47]^ This includes promoting inflammation, exacerbating RPE damage, and fostering CNV in wet AMD. The polarization of macrophages into pro-inflammatory (M1) and anti-inflammatory (M2) phenotypes and their role in tissue repair, regeneration, and fibrosis highlights the complexity of their involvement in AMD. Notably, the interplay between M1 and M2 macrophages and their impact on CNV development underscores the nuanced understanding required to elucidate their contribution to AMD.^[48,49]^ The activities of macrophages and microglia in AMD are influenced by a variety of signals from the retinal microenvironment, including oxidative stress, complement activation, and the presence of damaged cellular components. The balance between their protective and detrimental roles is crucial in determining the outcome of their involvement in AMD.^[4]^

Understanding the precise mechanisms by which macrophages and microglia contribute to AMD’s onset and progression is an area of active research. This includes identifying specific triggers that shift their roles from protective to detrimental, understanding the impact of aging on their function, and elucidating the molecular pathways that mediate their involvement in AMD pathogenesis. Moreover, exploring therapeutic strategies aimed at modulating their activity presents a promising avenue for AMD treatment. This could involve targeting specific signaling molecules or pathways to suppress their pro-inflammatory actions or enhance their tissue repair capabilities. Ultimately, a deeper understanding of microglia and macrophage biology in the context of AMD could pave the way for innovative interventions to halt or even reverse the progression of this debilitating disease.

#### 4.7.4. Autophagy in AMD

The prominence of autophagy, as indicated by its frequent occurrence in author keywords and its emergence in thematic evolution analysis, underscores its growing importance in understanding AMD’s pathogenesis. This aligns with the broader shift in AMD research towards exploring cellular and molecular mechanisms underpinning the disease, particularly those related to inflammation and cellular homeostasis.

Autophagy, a cellular process for the degradation and recycling of damaged organelles and proteins, serves as a protective mechanism that maintains cellular integrity within the RPE and other retinal cells by removing deleterious components that could trigger inflammatory responses. Dysregulation of autophagy has been implicated in the pathogenesis of AMD. Specifically, impaired autophagy in the RPE can lead to the accumulation of waste products, such as lipofuscin and extracellular drusen, which are hallmark features of AMD. These accumulations can exacerbate oxidative stress and inflammation, further contributing to the degenerative process.^[22,50]^

The exploration of therapeutic agents such as metformin or trehalose, known to modulate autophagy, opens promising avenues for AMD treatment. Their ability to restore autophagic function and counteract AMD-like phenotypes in experimental models underscores the therapeutic potential of autophagy modulation.^[51]^ Concurrently, the role of zinc in stimulating autophagy and its impact on AMD progression invites further investigation. Given zinc’s capacity to influence autophagic activity and potentially slow AMD progression, understanding the interplay between zinc supplementation, genetic predispositions, and environmental factors becomes crucial. This includes assessing the broader implications of zinc and nutritional factors on AMD, taking into account the genetic diversity and environmental variability among AMD patients.^[52]^

Future research should prioritize unraveling the diurnal regulation of autophagy and its implications for AMD pathology. The noted diurnal rhythmicity in the expression of autophagy-related proteins and its dysregulation in disease states suggest that temporal aspects of autophagy could offer new insights into therapeutic timing and efficacy. An integrative research approach, encompassing genetic, molecular, and environmental factors, will be essential in bridging the gap between basic science and clinical application. Investigating the complex interplay between autophagy, lysosomal function, and the broader cellular environment in AMD will pave the way for novel therapeutic strategies, offering hope for improved management and understanding of this prevalent condition.

### 4.8. Study limitations

All bibliometric analyses, including this study on inflammation in AMD, carry inherent limitations. Firstly, our dataset was exclusively derived from the WoSCC, a common yet selective source for bibliometric studies. This focus means relevant articles available in other databases (e.g., Scopus, PubMed) or published in languages other than English might not have been included, potentially skewing the breadth of our analysis. Furthermore, our analysis is subject to citation bias, wherein highly cited publications may disproportionately influence trends, potentially overlooking emerging or niche research. Additionally, our reliance on bibliometric software, which employs machine learning algorithms for data analysis, introduces a risk of analytical bias due to algorithmic limitations or interpretative errors. Another limitation stems from the publication delay inherent to academic research; new studies continuously emerge, and those published close to our analysis cutoff date may not have been included. This time lag means our study may miss the latest advancements or shifts in research focus within the AMD field, particularly concerning inflammation’s role.

Despite these constraints, our investigation endeavors to provide a comprehensive and systematic overview of the prevailing research trends, key contributors, and future directions within the inflammation in AMD domain. By mapping the landscape of this critical area of AMD research, we aim to furnish researchers with valuable insights, fostering further inquiry and potential collaborations in understanding and addressing this complex condition.

## 5. Conclusion

Leveraging information visualization technology, this study comprehensively delineates the evolution, focal points, and leading edges of research within the inflammation in AMD domain. Our findings lay a groundwork that could catalyze future inquiries in this sphere, fostering collaborations among researchers and institutions. The study reveals a robust growth trajectory in inflammation in AMD research, with significant contributions and potential collaborative opportunities present at institutions like the NEI and the University of Eastern Finland. The United States and China emerge as dominant players, steering the research direction and investments in this field. Within the academic landscape, Investigative Ophthalmology and Visual Science and Experimental Eye Research are identified as pivotal publication venues, with Kaarniranta among the most influential authors. This collaborative network spanning countries, institutions, and researchers has been instrumental in propelling the field forward. Our bibliometric analysis pinpoints oxidative stress, the complement system, autophagy and the roles of microglia/macrophages as current research hotspots. The interplay between the complement system’s dysregulation and AMD pathogenesis, alongside the dual roles of microglia/macrophages, underscores the complexity of inflammatory mechanisms in AMD. Future directions highlighted by our study include a deeper exploration of the complement system’s therapeutic targeting, elucidating microglia/macrophage activity’s nuances, and the critical impact of oxidative stress and autophagy on AMD.

## Author contributions

**Conceptualization:** Fangfang Lu, Zhi Wang.

**Data curation:** Fangfang Lu.

**Formal analysis:** Fangfang Lu.

**Funding acquisition:** Fangfang Lu, Jia Zhang, Qiguo Xiao.

**Investigation:** Fangfang Lu, Jia Zhang, Jing Lu, Weijie Jiang.

**Methodology:** Fangfang Lu, Jing Lu, Weijie Jiang.

**Project administration:** Zhi Wang.

**Supervision:** Zhi Wang.

**Validation:** Jia Zhang, Zhigang Fei, Qiguo Xiao, Zhi Wang.

**Visualization:** Fangfang Lu, Jia Zhang, Zhigang Fei.

**Writing – original draft:** Fangfang Lu, Jia Zhang, Jing Lu, Weijie Jiang.

**Writing – review & editing:** Zhigang Fei, Zhi Wang.

## Supplementary Material


