# Supplementary material for: Trends, hotspots, and future directions of inflammation in age-related macular degeneration: A 20-year bibliometric study
Source: Medicine (Baltimore). 2026 Mar 20;105(12):e46598. doi: 10.1097/MD.0000000000046598 (PMC13008227; doi:10.1097/MD.0000000000046598)

**Supplementary Table 1**. The top 10 cited publications related to inflammation in AMD.

| Rank | Title | First author | Journal | Publication year | Total citations |
| --- | --- | --- | --- | --- | --- |
| 1 | Current concepts in the pathogenesis of age-related macular degeneration (DIO:10.1001/archopht.122.4.598) | Zarbin, MA | ARCH OPHTHALMOL-CHIC | 2004 | 806 |
| 2 | The Role of the Reactive Oxygen Species and Oxidative Stress in the Pathomechanism of the Age-Related Ocular Diseases and Other Pathologies of the Anterior and Posterior Eye Segments in Adults (DIO:10.1155/2016/3164734) | Nita, M | OXID MED CELL LONGEV | 2016 | 687 |
| 3 | Complement system part II: role in immunity (DIO:10.3389/fimmu.2015.00257) | Merle, NS | FRONT IMMUNOL | 2015 | 669 |
| 4 | Understanding age-related macular degeneration (AMD): Relationships between the photoreceptor/retinal pigment epithelium/Bruch's membrane/choriocapillaris complex (DIO:10.1016/j.mam.2012.04.005) | Bhutto, I | MOL ASPECTS MED | 2012 | 656 |
| 5 | The pivotal role of the complement system in aging and age-related macular degeneration: Hypothesis re-visited (DIO:10.1016/j.preteyeres.2009.11.003) | Anderson, DH | PROG RETIN EYE RES | 2010 | 561 |
| 6 | Oxidative damage-induced inflammation initiates age-related macular degeneration (DIO:10.1038/nm1709) | Hollyfield, JG | NAT MED | 2008 | 548 |
| 7 | Does neuroinflammation fan the flame in neurodegenerative diseases? (DIO:10.1186/1750-1326-4-47) | Frank-Cannon, TC | MOL NEURODEGENER | 2009 | 544 |
| 8 | An animal model of age-related macular degeneration in senescent Ccl-2-or Ccr-2-deficient mice (DIO:10.1038/nm950) | Ambati, J | NAT MED | 2003 | 500 |
| 9 | Para-inflammation in the aging retina (DIO:10.1016/j.preteyeres.2009.06.001) | Xu, HP | PROG RETIN EYE RES | 2009 | 483 |
| 10 | Drusen complement components C3a and C5a promote choroidal neovascularization (DIO:10.1073/pnas.0408835103) | Nozaki, M | P NATL ACAD SCI USA | 2006 | 481 |

**Supplementary Figure 1.** **Flowchart of literature selection.** The diagram illustrates the process of identifying and screening publications from the Web of Science Core Collection database, leading to the final inclusion of 2,044 articles for analysis


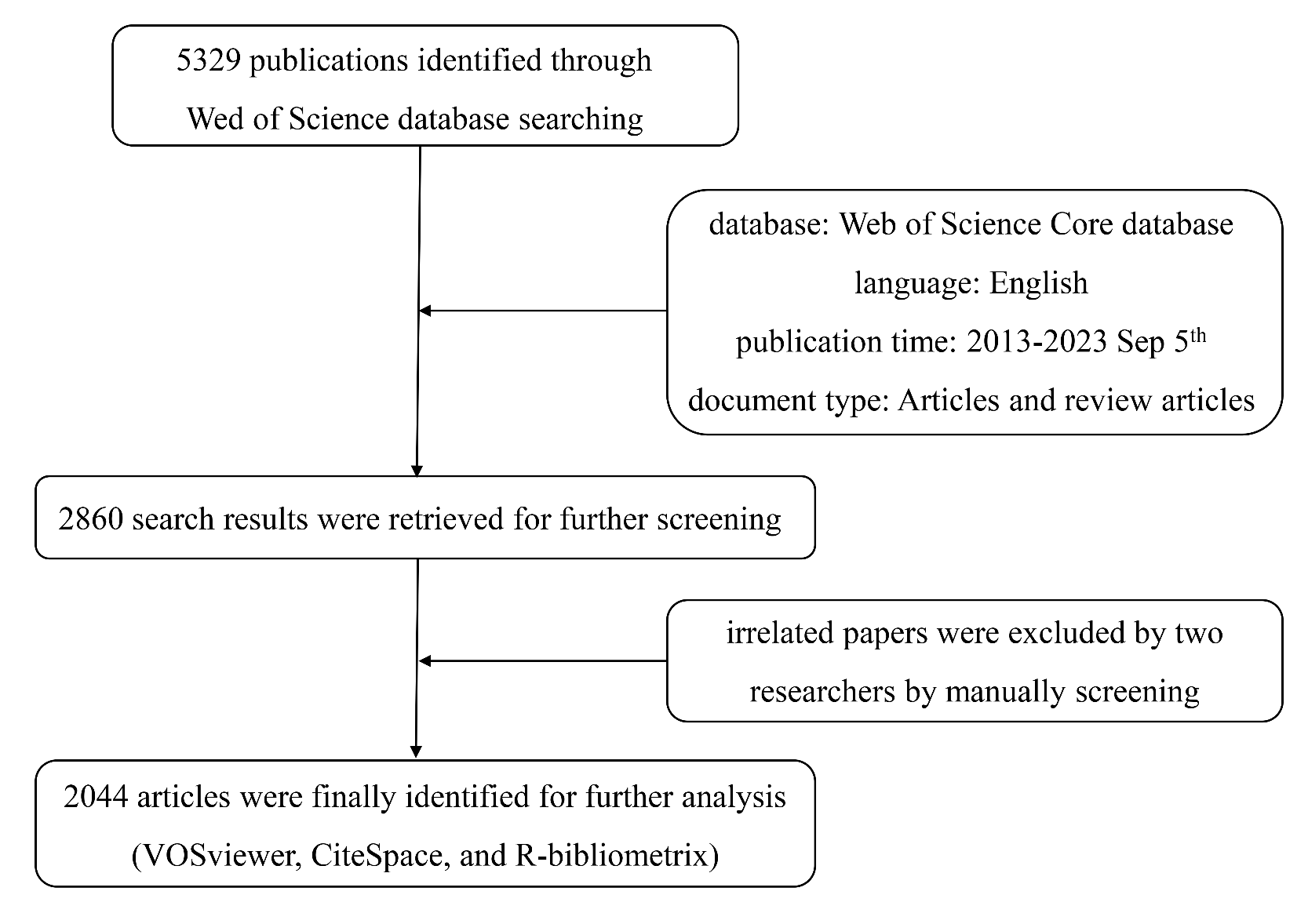

Supplement: Supplementary file 1 [file medi-105-e46598-s001.docx]
